# The competitive interplay of 12‐oxophytodienoic acid (OPDA), protein thiols, and glutathione

**DOI:** 10.1111/febs.70436

**Published:** 2026-02-05

**Authors:** Madita Knieper, Ruben Schwarz, Lara Vogelsang, Jens Sproß, Armağan Kaya, Maike Bittmann, Harald Gröger, Andrea Viehhauser, Karl‐Josef Dietz

**Affiliations:** ^1^ Biochemistry and Physiology of Plants, Faculty of Biology Bielefeld University Germany; ^2^ Industrial Organic Chemistry and Biotechnology, Faculty of Chemistry Bielefeld University Germany; ^3^ Department of Engineering Basic Sciences, Faculty of Engineering Alanya Alaaddin Keykubat University Turkey

**Keywords:** 12‐oxophytodienoic acid, cyclophilin 20–3, *retro*‐Michael addition, thiol‐disulfide exchange, thioredoxin

## Abstract

*Cis*‐(+)‐12‐oxophytodienoic acid (OPDA) is a bioactive oxylipin and phytohormone participating in regulation of plant stress responses, growth, and development. Due to its α, β‐unsaturated carbonyl moiety, OPDA covalently binds to free thiol groups by Michael addition. This binding, termed OPDAylation, alters the activity of target proteins, such as cyclophilin 20‐3 (EC:5.2.1.8) and thioredoxins, that are essential components of the cellular redox regulatory network. To function as a reversible redox regulatory mechanism, OPDAylation should be complemented by a process of de‐OPDAylation allowing for fine‐tuning of OPDA‐dependent regulation. This study explored OPDAylation and de‐OPDAylation *in vitro* with emphasis on the involvement of glutathione. OPDA can be transferred from protein to glutathione (GSH) and *vice versa*. In a competition experiment, OPDAylation of thioredoxins (TRX) occurred rapidly in the presence of GSH, while over extended incubation times, de‐OPDAylation of TRX occurred due to the stoichiometric excess of GSH. These results support the hypothesis that the initial TRX‐based OPDAylation is proceeding under kinetic control due to the higher reactivity of the more nucleophilic cysteine moiety in TRX compared to the one of GSH, while the OPDAylation of GSH observed at prolonged incubation time is then the result of a thermodynamically controlled process. De‐OPDAylation depends on the protein's sensitivity towards OPDA, the pH and the concentration of excess thiol groups. This likely allows for precise modulation of OPDA amounts, as the rapid modification of protein activity enables subsequent induction of OPDA signaling, whereas de‐OPDAylation, triggered by increasing glutathione, increasing cellular reduction or presumably enzymatically, reverses this effect.

## Graphical abstract

12‐Oxophytodienoic acid (OPDA) is a phytohormone involved in plant growth and stress defense. Due to its cyclopentenone moiety, OPDA can form Michael adducts with thiol‐containing compounds such as glutathione and cysteine residues of proteins, resulting in alterations of the cellular redox regulatory network. Our findings reveal transferability of OPDA between OPDA adducts, indicating a novel regulatory mechanism of OPDA signaling which allows for fine‐tuning of OPDA signaling.

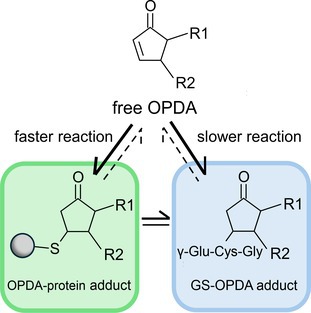

**Abbreviations**
4‐HNE: 4‐hydroxynonenal
ABC transporter: ATP‐binding cassette transporter
ANOVA: Analysis of variance
AOC: allene oxide cyclase
AOS: allene oxide synthase
CSC: cysteine synthase complex
Cyp20‐3: cyclophilin 20–3
DHAR: dehydroascorbate reductase
dn‐OPDA: dinor‐OPDA
DTNB: 5,5'‐dithiobis‐2‐nitrobenzoic acid
DTT: dithiothreitol
EEE: excess excitation energy
GEO: Gene Expression Omnibus
GGT: γ‐glutamyl transpeptidase
GPXL: glutathione peroxidase‐like
GSH: glutathione
GST: glutathione‐*S*‐transferase
JA: jasmonic acid
KPi: potassium phosphate
LOX: lipoxygenase
MRP: multidrug resistance‐associated protein
MS: mass spectrometry
NCBI: National Center for Biotechnology Information
NTRA: NADPH‐dependent thioredoxin reductase A
OPC:8–0: 8‐(3‐oxo‐2‐(pent‐2‐enyl)cyclopentenyl)octanoid acid
OPDA: *Cis*‐(+)‐12‐oxophytodienoic acid
OPR: OPDA reductase
ORG: OPDA‐responsive gene
PPIase: peptidyl‐prolyl‐*cis*‐*trans*‐isomerase
PTM: post‐translational modification
r.u.: relative units
RCS: reactive carbonyl species
ROS: reactive oxygen species
TRX: thioredoxin
WT Col‐0: wild‐type Columbia‐0
α‐LeA: α‐linolenic acid

## Introduction

*Cis*‐(+)‐12‐oxophytodienoic acid (OPDA) is a cyclopentenone oxylipin, which is synthesized in the chloroplast from α‐linolenic acid by 13‐lipoxygenase (13‐LOX, EC:1.13.11.12), allene oxide synthase (AOS, EC:4.2.1.92), and allene oxide cyclase (AOC, EC:5.3.99.6) [1]. While it is a precursor for the plant hormone jasmonic acid (JA), OPDA itself acts as a phytohormone affecting crucial processes such as germination, plant growth, and stress defense [2, 3, 4, 5, 6].

OPDA bioactivity originates from its cyclopentenone moiety which assigns it to the group of reactive carbonyl species (RCS) as a highly reactive α, β‐unsaturated carbonyl compound that forms covalent adducts with free thiol groups by Michael addition [7, 8, 9]. Formation of protein OPDA adducts by Michael addition, termed OPDAylation, was previously described as a novel posttranslational modification (PTM) affecting thiol switch proteins like thioredoxins (TRX) and, thereby, modulating the redox regulatory network of plant cells [10, 11, 12].

The most prominent target of OPDAylation in *Arabidopsis* is cyclophilin 20–3 (Cyp20‐3). Formation of Cyp20‐3‐OPDA adducts enhances formation of the cysteine synthase complex (CSC), activating thiol synthesis and subsequently increasing the cellular reduction potential. This increase, in turn, modulates the expression of OPDA‐responsive genes (ORGs) that contribute, for example, to plant defense under wounding or high light stress [11, 13].

High light stress is a frequently occurring abiotic stress condition that is initiated if the photosynthetic active radiation exceeds the growth light, and in full sunlight reaching > 2000 μmol photons m^−2^·s^−1^ in temperate latitudes [14, 15]. Excess excitation energy (EEE) enhances the production of reactive oxygen species (ROS) such as H_2_O_2_ and O_2_^–^ in the chloroplast. To cope with EEE, defense mechanisms are needed to adjust ROS scavenging and maintain cellular redox balance [16, 17]. In this context, adjustment of gene expression occurs within seconds [18]. Recent studies positioned OPDA as the responsible jasmonate coordinating expression of stress defense genes under early stage EEE stress with JA‐mediating plant recovery only afterwards [17, 19, 20]. Moreover, OPDA‐specific signaling presumably also extends to long‐term high light stress, as indicated by the uncoupling of OPDA and JA synthesis [3, 21]. At this point, OPDA‐mediated photoprotection traces back to enhanced ROS scavenging and chaperone capacities as well as synthesis of nonphotosynthetic pigments like flavonoids and glucosinolates [11, 17, 19, 20, 22, 23, 24].

Nevertheless, as RCS like OPDA accumulate, they can cause oxidative damage to proteins, lipids, and DNA [7, 22, 25, 26, 27]. Therefore, tight regulation of OPDA levels is essential. Recently, conjugates of OPDA and various amino acids have been discovered in *A. thaliana* [28, 29, 30] and rice [31]. In contrast to JA‐isoleucine, these conjugates, like dn‐OPDA‐amino acid‐conjugates [32], might be inactive compounds contributing to the homeostasis of OPDA levels as OPDA storage pools [30].

Furthermore, detoxification of OPDA occurs by adduct formation (GS‐OPDA) with glutathione (GSH), the major low molecular mass thiol antioxidant in eukaryotes, and subsequent vacuolar degradation (catalyzed by γ‐glutamyl transpeptidase 4 [GGT4, EC:2.3.2.2]) [27, 33]. Various glutathione‐*S*‐transferases (GSTs) of the phi and tau class have been attributed to GS‐OPDA formation, with GST activities towards OPDA ranging from 0.1–0.3 nkat mg^−1^ (GSTU1, GSTU2, GSTU4, GSTU5) and 0.4–0.8 nkat mg^−1^ (GSTU8, GSTU19) to 1.0–1.6 nkat mg^−1^ (GSTF8, GSTU6, GSTU10, GSTU17, GSTU25) [34, 35].

In general, to efficiently participate in dynamic regulation, reversibility of a particular PTM is an important prerequisite. Although Michael addition is widely regarded as a relatively stable process with low reversibility [36], reversibility of RCS coupling contributes to fine‐tuning of RCS signaling as has been shown for 4‐HNE, acrolein, prostaglandins, and isoprostanes [37, 38, 39, 40, 41].

Since recovery of OPDAylated proteins has not been studied so far, this investigation was designed to explore whether OPDA‐thiol adducts might undergo *retro*‐Michael addition, allowing for (a) improved fine‐tuning of OPDA signaling under stress conditions and (b) potential recovery of free OPDA after adduct formation.

The latter would be of special interest as it might explain how a free OPDA pool for OPDA signaling and conversion to JA or OPDA‐amino acid adducts is maintained in the presence of high chloroplastic and cytosolic thiol levels.

Furthermore, this study gives an insight into the competing roles of cysteine‐containing moieties in the formation of OPDAylated Michael‐type products, exemplified for TRX and GSH as *S*‐nucleophiles.

Our work addresses this dilemma *in vitro* by employing TRXs and Cyp20‐3 as selected target proteins of OPDAylation. Additionally, H_2_O_2_ and flavonoid levels of *A. thaliana* leaf discs exposed to EEE grant insight to OPDA signaling and its possible reversibility *in vivo*. The results reveal a dynamic network of OPDA, protein OPDA adducts, and GS‐OPDA, hypothetically dependent on variation of GST expression.

## Results

OPDA signaling function has been experimentally linked to a variety of stress defense mechanisms in plants. However, so far it remained unknown whether OPDAylation is a permanent protein modification and how it can be achieved despite the high prevalence of GSH under physiological conditions. To this end, the aim of this work was to explore the effect of excess free thiols on the formation of OPDA adducts as well as the reversibility of OPDAylation (de‐OPDAylation).

### Formation of OPDA adducts

**Fig. 1:**
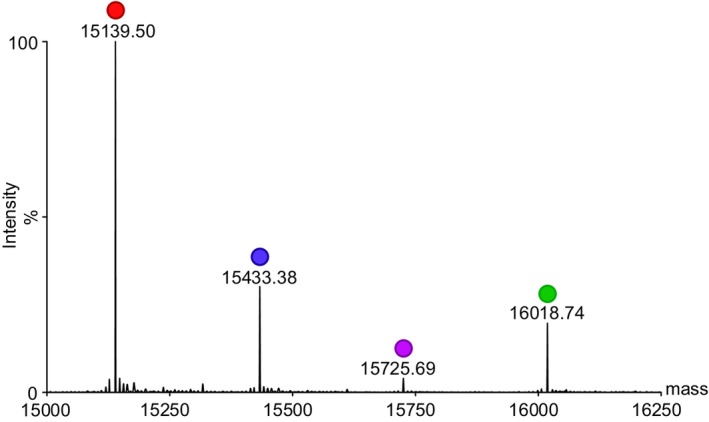
Mass spectrometric analysis of adduct formation between thioredoxin (TRX)‐h3 and 12‐oxophytodienoic acid (OPDA). 20 μm protein was incubated with 80 μm OPDA in 50 mm ammonium acetate for 16 h prior to measurement. Different charge states of the protein could be detected revealing free (native) TRX (15.14 kDa, red dot) and 1x (15.43 kDa, blue dot), 2x (15.73 kDa, purple dot) or 3x OPDAylated protein (16.02 kDa, green dot).

As a first step, generation of OPDA adducts was studied under control conditions to confirm the reactivity of OPDA towards thiol groups. TRX‐h3 was chosen as a target protein since it is highly susceptible to OPDAylation [10]. When testing various stoichiometric ratios, a 4 : 1 OPDA:TRX‐h3 ratio yielded fully OPDAylated protein after 16 h of incubation (Fig. 1 and Table 1). Hence, this ratio was adopted for MS analysis of OPDAylated TRX.

**Table 1:** Mass spectrometric analysis of thioredoxin (TRX)‐h3‐12‐oxophytodienoic acid (OPDA) adducts using different OPDA:protein ratios. 20 μm of recombinant protein was incubated with 20–80 μm of OPDA at RT for 16 h. Mass is given in Dalton ± standard deviation. Colored dots refer to the individual peaks presented in Fig. 1.

OPDA:TRX ratio	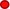 Base peak	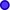 Peak 1	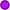 Peak 2	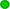 Peak 3
0 : 1	15140.7 ± 0.0	‐	‐	‐
1 : 1	15139.3 ± 0.0	15433.5 ± 0.0	15725.6 ± 0.2	‐
2 : 1	15139.4 ± 0.0	15433.6 ± 0.0	15725.9 ± 0.1	‐
3 : 1	15139.4 ± 0.0	15433.6 ± 0.1	15 726 ± 0.1	‐
4 : 1	15139.5 ± 0.1	15433.4 ± 0.1	15725.7 ± 0.1	16018.7 ± 0.3

**Fig. 2:**
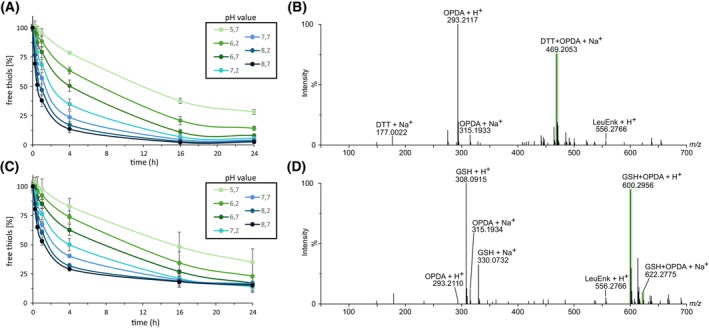
Formation of dithiothreitol‐12‐oxophytodienoic acid adducts (DTT‐OPDA) and glutathione‐OPDA adducts (GS‐OPDA). OPDA (500 μm) was incubated with 250 μm DTT (A, B) or GSH (C, D) in 40 mm KPi, pH 5.7–8.7, and residual free thiols were quantified at indicated time points. Data are means ± SD of *n* = 3 independent replicates. (A, C) Alternatively, 150 μm OPDA was incubated with 1 mm GSH or DTT overnight and subjected to mass spectrometry (B, D). Labeled arrows indicate the masses of specified molecular species and adducts of DTT and OPDA and GSH and OPDA.

**Fig. 3:**
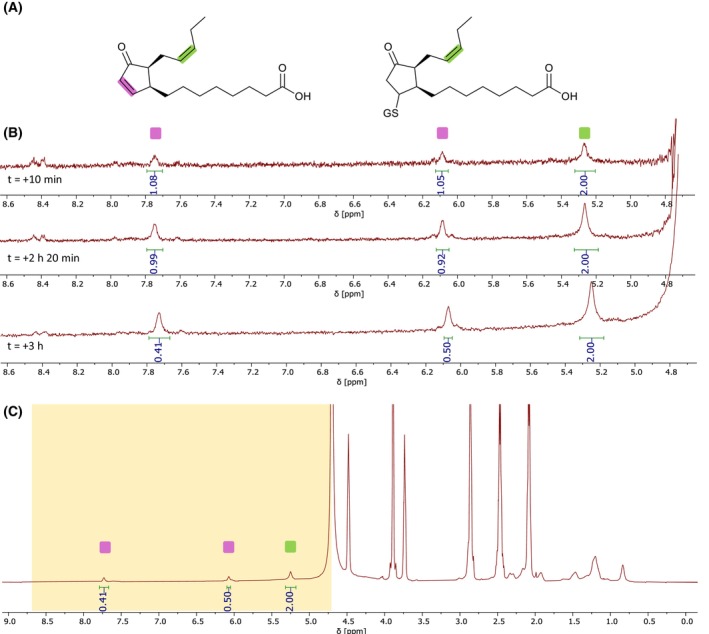
Structures of 12‐oxophytodienoic acid (OPDA) and OPDA‐glutathione (GSH) adducts (GS‐OPDA). The double‐bond protons of the cyclopentenone ring and the α‐side chain have been marked in pink and green, respectively (A). Comparison of ^1^H‐nuclear magnetic resonance (NMR) spectra taken at different time points (B). The signals are assigned by color. The formation of GS‐OPDA is indicated by the disappearance of the signals of the cyclopentenone double bond. Full ^1^H‐NMR‐spectrum recorded at timepoint *t* = + 3 h (C). The zoomed‐in section from (B) is highlighted in yellow.

Binding of OPDA to nonprotein thiols, namely GSH and DTT, could be observed by a gradual decrease of free thiol groups and formation of the respective adducts (Fig. 2A–D). Furthermore, thiol quantification revealed a pH‐dependent adduct formation, presumably by altering the protonation state of the thiol group (Fig. 2A,C). Additional analysis by means of ^1^H‐NMR spectroscopic studies confirmed a conversion of 59% over an incubation time of 3 h (calculated on the basis of the ^1^H‐NMR‐signals of the C*H*‐C(O)‐proton at 7.73 ppm and the two C*H*=C*H*‐protons at 5.25 ppm) (Fig. 3A–C).

### Disintegration of protein adducts by excess thiols

As OPDA adducts proved to be stable under control conditions, GSH was added to OPDAylated TRX‐h3, and possible reversibility of OPDAylation was assessed by MS analysis. GSH was chosen as a potential trigger of de‐OPDAylation as it is the dominant low molecular mass thiol antioxidant in the cell and was previously reported to support *retro*‐Michael addition of TRX‐acrolein adducts [38].

**Fig. 4:**
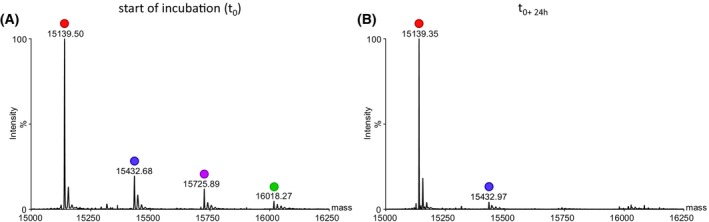
Time‐dependent de‐OPDAylation of thioredoxin (TRX)‐h3 after addition of glutathione (GSH). Recombinant TRX was reduced, desalted, treated with 12‐oxophytodienoic acid (OPDA) for 16 h and desalted again. Samples were subjected to mass spectrometric analysis after addition of GSH at 0 h (A) or 24 h (B). Peaks correspond to free TRX (15.14 kDa, red dot) and 1‐3x OPDAylated TRX (15.43 kDa and blue dot, 15.73 kDa and purple dot, 16.02 kDa and green dot, respectively).

Obtained spectra, resembling those depicted in Fig. 1, showed a slow gradual decrease of TRX‐OPDA adducts after addition of GSH (Fig. 4A,B). The peaks corresponding to twofold and threefold OPDAylated protein vanished almost completely after 24 h.

**Fig. 5:**
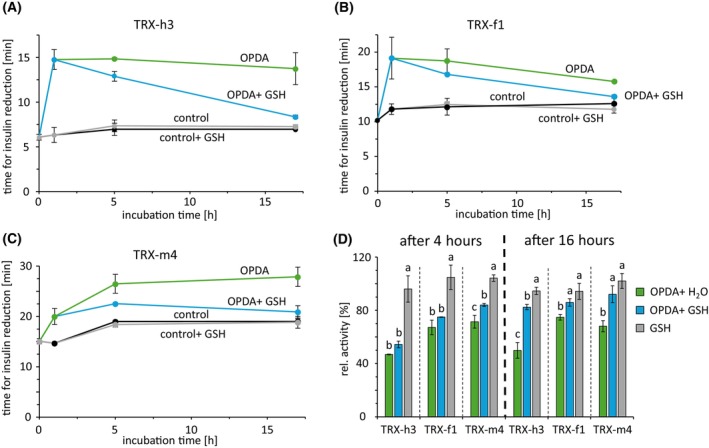
Insulin reduction activity of thioredoxins (TRXs) after (de)‐OPDAylation. Recombinant TRX (20 μm) was treated with 12‐oxophytodienoic acid (OPDA, 80 μm) or 0.27% (v/v) EtOH as mock control for 1 h. Then, glutathione (GSH), or water as mock control, was added and activity was determined after 4 and 16 h using TRX at a final concentration of 5 μm. (A–C) Protein activity is depicted as the time needed to pass the threshold of ΔA_650 nm_ = 0.3. (D) TRX activity was determined as the change in A_650 nm_ min^−1^ and normalized to the control (mock‐treated sample, 100% activity equals ∆A_650nm_ min^−1^ = 0.045, 0.025, and 0.016 (t4) and 0.045, 0.027, and 0.016 (t16) for TRX‐h3, ‐f1, and ‐m4, respectively. Significance of difference was assessed in each TRX assay, respectively, by one‐way ANOVA and Tukey test (*P* < 0.05) and is denoted as letters a‐c. Data in all panels are means ± SD of *n* = 3 (t16: TRX‐f1 + OPDA and TRX‐f1 + EtOH+GSH), *n* = 4 (t1: TRX‐h3 + OPDA, TRX‐m4 + EtOH, t5: TRX‐h3 + OPDA, all TRX‐f1 samples, TRX‐m4+ EtOH, TRX‐m4 + EtOH+GSH, t16: TRX‐h3 + OPDA, TRX‐h3/m4 + EtOH), *n* = 5 (all t0 samples, t1: TRX‐f1 + OPDA, TRX‐f1 + EtOH, t5: TRX‐h3 + OPDA+GSH, TRX‐m4 + OPDA, TRX‐m4 + OPDA+GSH, t16: TRX‐h3/f1/m4 + OPDA+GSH, TRX‐h3/m4 + EtOH+GSH, TRX‐f1 + EtOH, TRX‐m4 + OPDA) or *n* = 6 (t1: TRX‐h3 + EtOH, TRX‐m4 + OPDA, t5: TRX‐h3:EtOH, TRX‐h3 + EtOH+GSH) independent replicates.

**Fig. 6:**
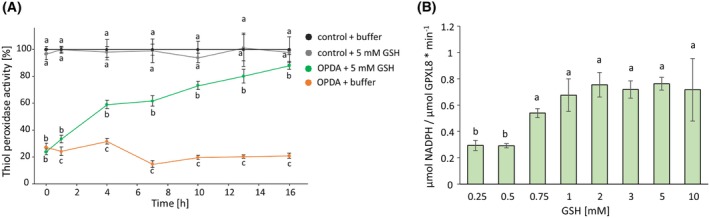
Thioredoxin (TRX)‐dependent H_2_O_2_ reduction activity of Glutathione peroxidase‐like (GPXL) 8. Following OPDAylation or mock treatment (0.5% [v/v] EtOH) of TRX‐h3 for 1 h, glutathione (GSH), or an equivalent amount of water as control, was added and GPXL8 activity with TRX‐h3 as electron donor was determined using the NADPH**→**NTRA**→**TRX‐h3**→**GPXL8**→**H_2_O_2_ reconstitution assay. Mean and SD for *n* = 3 (TRX + EtOH, TRX + EtOH+GSH) or *n* = 6 (TRX + OPDA, TRX + OPDA+GSH) independent replicates are shown (A). Recovery of GPXL activity after 16 h was further assessed in the presence of varying GSH concentrations (0.25–10 mm). Data are means ± SD of *n* = 3 (0.25 and 0.5 mm GSH) or *n* = 6 (all others) independent replicates (B). Significances of difference between the different samples at a given time point were determined using ANOVA, followed by post hoc Tukey test and are marked with letters a–c.

The observed decrease of TRX‐OPDA adducts hinted towards a reversibility of OPDAylation by excess free thiols; however, the oxidation state and/or activity of released protein remained unclear. To address this question, protein activity was measured analogously to previous studies [11]. The insulin reduction capacity of different thioredoxins (TRX‐h3, ‐f1 and ‐m4) was chosen as a general readout for disulfide reduction activity (Fig. 5A–D) and TRX‐dependent GPXL8 activity as a specific reduction assay (Fig. 6A,B). OPDAylation showed an inhibitory effect on all tested TRXs. The residual activity was 47% for TRX‐h3, 67% for TRX‐f1, and 71% for TRX‐m4. Both the insulin assay and the GPXL8 assay revealed recovery of the reductase activity after addition of 1 mm GSH (Figs 5 and 6).

To determine the protein redox state after de‐OPDAylation, intrinsic tryptophan fluorescence of TRX‐f1 and TRX‐m4 was measured (Table 2). After OPDAylation, relative fluorescence intensity of both proteins was quenched (–14.5 and –16.3 for TRX‐f1 and ‐m4, respectively) to a similar level as oxidized protein. This decrease was more pronounced after longer incubation. After the addition of GSH, fluorescence intensity of both TRXs increased and almost reached the value of reduced protein. It is concluded that TRX‐f1 and ‐m4 returned to the reduced state after the OPDA release. This would be in accordance with the return of protein activity as thioredoxins are redox‐sensitive proteins.

**Table 2:** Intrinsic protein fluorescence of 15 μm thioredoxin (TRX)‐f1 and TRX‐m4 incubated with 12‐oxophytodienoic acid (OPDA) ± glutathione (GSH). Given values refer to maximal fluorescence intensity (relative units). Data are means ± SD of *n* = 5. Statistically significant differences (one‐way ANOVA and post hoc Dunnett test, *P* < 0.05) are indicated by *.

Incubation time time	Treatment	TRX‐f1	TRX‐m4
3 h	Control	116.8 ± 1.9	115.3 ± 3.1
	OPDA	102.3 ± 1.8 *	99.0 ± 1.7 *
	H_2_O_2_	99.9 ± 1.3 *	98.2 ± 1.9 *
24 h	Control + H_2_O	118.6 ± 3.1	118.7 ± 3.0
	Control + GSH	114.8 ± 3.5	117.9 ± 2.9
	OPDA + H_2_O	87.7 ± 2.9 *	91.6 ± 3.3 *
	OPDA + GSH	106.2 ± 3.1 *	105.5 ± 2.5 *

**Fig. 7:**
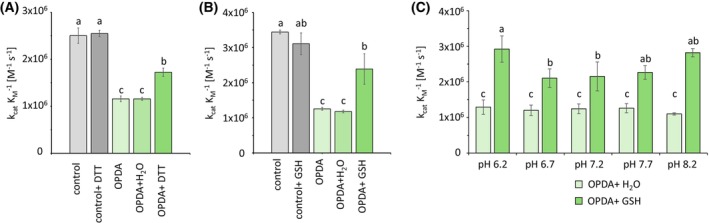
Recovery of peptidyl‐prolyl‐*cis*‐*trans*‐isomerase (PPIase) activity of OPDAylated Cyclophilin 20‐3 (Cyp20‐3) by glutathione (GSH) or dithiothreitol (DTT). Pretreatment of 20 μm Cyp20‐3 with 150 μm 12‐oxophytodienoic acid (OPDA) inhibited PPIase activity. This inhibition was partially reversed upon incubation in the presence of 1 mM GSH or DTT as determined after 10 min (A), 48 h (B), or 4 h (C) of incubation. Data are means ± SD of *n* = 6 (samples at pH 7.2) or *n* = 3 (all other samples) independent replicates. Significant differences (*P* < 0.05, marked as a–c) were calculated by one‐way ANOVA and Tukey test.

Binding of OPDA to Cyp20‐3 stimulates thiol synthesis by stabilizing the Cys synthase complex. This effect is a central mechanism of OPDA‐specific signaling [11]. Therefore, the next experiments addressed OPDAylation of Cyp20‐3. After 10 min of OPDA treatment, PPIase activity of Cyp20‐3 decreased drastically (Fig. 7A,B), which is in accordance with previous studies [10, 42]. As expected, the addition of DTT and GSH caused a partial return of catalytic efficiency (211 and 149% recovery). Like GS‐OPDA formation (Fig. 2C), the degree of reactivation depended on the pH value of the medium with higher recovery at pH 6.2 compared to pH 6.7 and 7.2 (Fig. 7C).

**Fig. 8:**
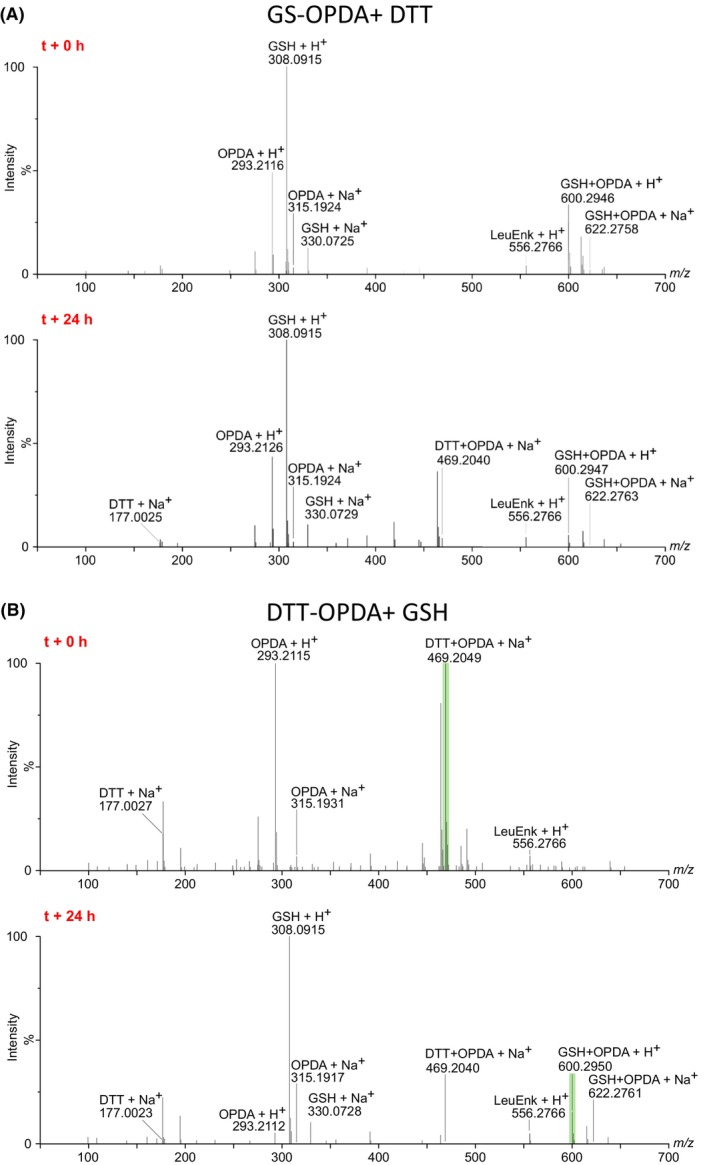
Competition between glutathione (GSH) and dithiothreitol (DTT) for formation of 12‐oxophytodienoic acid (OPDA) adducts. 150 μm OPDA was incubated with 1 mm GSH (A) or 1 mm DTT (B) for 16 h at RT. After addition of 1 mm DTT (A) or 1 mm GSH (B) and incubation for up to 24 h, formed adducts were assessed by mass spectrometry. Depicted are measurements after 0 and 24 h.

Lastly, release of OPDA from Michael adducts was studied by incubating GS‐OPDA with DTT (Fig. 8A) and DTT‐OPDA with GSH (Fig. 8B) and subsequent determination of OPDA adducts by MS. As expected, both DTT and GSH caused disintegration of adducts while forming new conjugates to OPDA. Interestingly, addition of GSH to DTT‐OPDA adducts caused complete vanishing of DTT‐adducts after 24 h, while GS‐OPDA adducts remained stable in the presence of DTT.

**Fig. 9:**
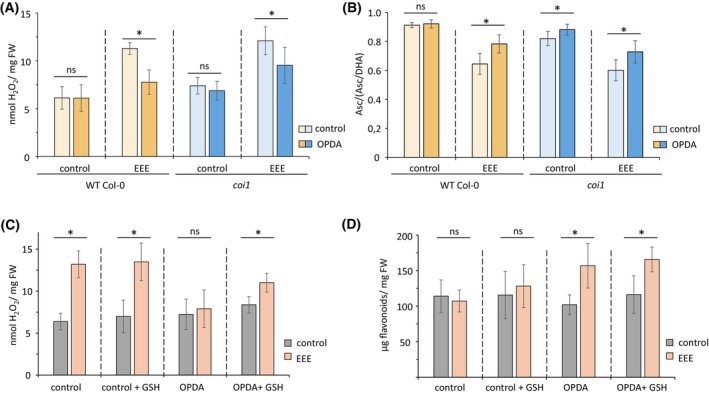
H_2_O_2_, ascorbate and flavonoid content of leaf discs exposed to high light stress for 6 h. Wild‐type (WT) plants were compared to *coi1*, which are impaired in jasmonic acid signaling perception (A, B). Effect of 25 μm 12‐oxophytodienoic acid (OPDA, dark colors) vs. mock control (light colors) was determined in regard to excess excitation energy (EEE)‐induced H_2_O_2_ accumulation (A) and ratio of reduced ascorbate (Asc) to total ascorbate (Asc + DHA, B). Data are means ± SD of *n* = 8. Based on these findings, leaf discs were treated with 25 μm OPDA, 0.27% (v/v) EtOH as control or 1 mm GSH in different combinations to assess the effect of GSH addition on OPDA signaling based on H_2_O_2_ (C) or flavonoid content (D). Statistical significance of differences (*P* < 0.05) was assessed by t‐test and are denoted as *. Data are means ± SD of *n* = 5 (EtOH without GSH at control conditions), *n* = 6 (OPDA at control conditions in panel D), or *n* = 8 (all others). Abbreviations: EEE: excess excitation energy; ns: insignificant.

To obtain first insights into de‐OPDAylation *in vivo*, leaf discs of *A. thaliana* were pre‐incubated with 25 μm OPDA, then 1 mm of GSH was added to the floating solution directly before exposure to EEE stress (800 μmol photons m^−2^·s^−1^, 6 h). This test system for OPDA effects on leaf discs had been optimized and characterized, also for its effects on the ascorbate and dehydroascorbate system of the tissue which is the electron donor to ascorbate peroxidases in H_2_O_2_ detoxification. The overnight incubation with OPDA for 16 h and the subsequent EEE treatment for 6 h reliably showed a suppression of the EEE‐induced H_2_O_2_ increase (Fig. 9A) with concomitant less oxidation of the ascorbate system (Fig. 9B). This effect was also seen in the *coi* mutant with defective JA signaling, indicating that this effect is upstream of JA, and, therefore, most likely a direct OPDA effect.

Addition of GSH to the solution directly before stress exposure partially hindered OPDA‐dependent signaling as indicated by a lesser increase in H_2_O_2_ (Fig. 9C). Additionally, measurement of total flavonoid content revealed basal flavonoid levels in mock‐treated samples, whereas OPDA pretreatment caused a significant increase (Fig. 9D). In contrast to H_2_O_2_ content, the addition of GSH did not lead to an inhibition of this readout of OPDA signaling.

Overall, addition of GSH to protein OPDA adducts regenerated the reduced and active protein form *in vitro*. Occurrence of de‐OPDAylation is further supported by GSH‐mediated suppression of OPDA‐dependent reduction in H_2_O_2_ levels after EEE stress. Since de‐OPDAylation was quite slow as compared to OPDAylation, enzymatic acceleration *in vivo*, for instance by GSTs, may be hypothesized.

### Does GSH function as an efficient competitor for protein OPDAylation?

As GSH majorly influences OPDA signaling by adduct formation as well as de‐OPDAylation, a possible antagonistic effect of GSH against OPDAylation of proteins might be assumed. In contrast to the abundant GSH present in mm concentrations, OPDA concentrations remain in the μm range even under stress. If GSH would act as a scavenger of OPDA *in vivo*, GS‐OPDA adducts should already form in the chloroplast and any OPDA signaling might be highly unlikely. Hence, our next experiments addressed the question whether OPDAylation of proteins occurs also under physiological concentrations of OPDA (μm) and GSH (mm).

**Fig. 10:**
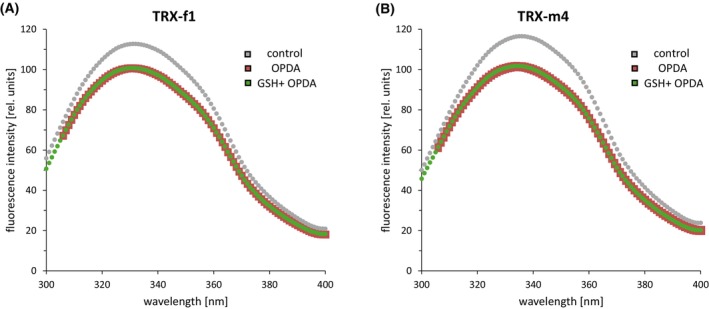
Intrinsic tryptophan fluorescence of thioredoxin (TRX)‐f1 and TRX‐m4. 15 μm TRX‐f1 (A) or TRX‐m4 (B) was incubated with 1 mm glutathione (GSH) for 2 h followed by 150 μm 12‐oxophytodienoic acid (OPDA) for 3 h. As a control, protein was incubated with GSH and 0.27% (v/v) EtOH. Data are means of *n* = 5. The red symbol size of OPDA treatment was set larger than the others because otherwise, they just disappeared behind the GSH + OPDA values.

Effects of OPDA on intrinsic fluorescence of TRX‐f1 and ‐m4 were measured in the presence of GSH (Fig. 10A,B and Table 3). As before, OPDA treatment led to a significant decrease in fluorescence intensity (−11.98 relative units (r.U.) and ‐14.84 r.U. for TRX‐f1 and m4, respectively), resembling the OPDA effect without GSH (Table 2) and arguing against a protective effect of GSH.

**Table 3:** Intrinsic protein fluorescence of 15 μm thioredoxin (TRX)‐f1 and TRX‐m4 incubated with 12‐oxophytodienoic acid (OPDA) without or with pretreatment with glutathione (GSH) for 2 h. Given values refer to maximal fluorescence intensity (relative units). Data are means ± SD of *n* = 5. Statistically significant differences (*P* < 0.05) of fluorescence intensity after OPDA treatment were calculated by one‐way ANOVA and post hoc Dunnett test against ‘control+EtOH’ and are denoted as *.

Treatment	TRX‐f1	TRX‐m4
control + EtOH	116.8 ± 1.9	115.3 ± 3.1
OPDA	102.3 ± 1.8 *	99.0 ± 1.7 *
GSH + EtOH	112.7 ± 0.4 *	116. 6 ± 0.7
GSH + OPDA	100.7 ± 0.7 *	101.7 ± 0.3 *

**Fig. 11:**
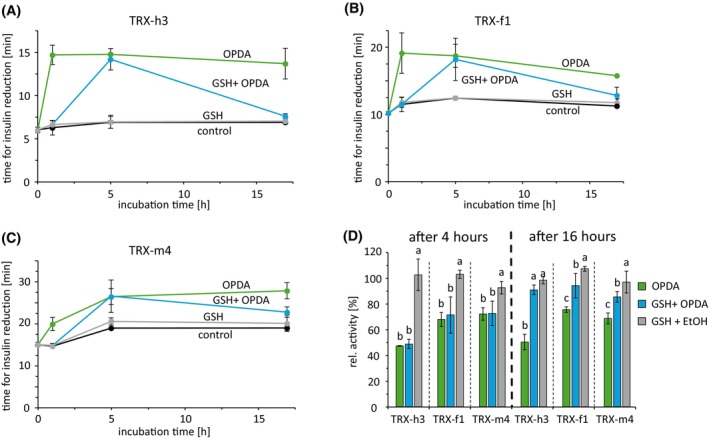
Insulin reduction activity of thioredoxins (TRXs) after pretreatment with glutathione (GSH). Recombinant TRX (20 μm) was treated with 1 mm GSH for 1 h. Then, 80 μm 12‐oxophytodienoic acid (OPDA) or 0.27% [v/v] EtOH were added, and activity was measured after 4 and 16 h (A–C). Protein activity is depicted as the time needed to pass the threshold of ΔA_650 nm_ = 0.3 (D). Thioredoxin activity was determined as the change in A_650 nm_ minute^−1^ using a final protein concentration of 5 μm. Data were normalized to a control sample containing protein incubated only with 0.27% [v/v] EtOH. Significance of difference was assessed in each TRX assay respectively by one‐way ANOVA and Tukey test (*P* < 0.05) and is marked by letters a‐c. Data in all panels are means ± SD of *n* = 3 (t5: TRX‐h3 + GSH + OPDA, t16: TRX‐f1 + OPDA), *n* = 4 (t1: TRX‐h3 + OPDA, TRX‐h3/m4 + GSH, TRX‐m4 + EtOH, t5: TRX‐h3/f1 + OPDA, TRX‐f1/m4 + GSH + OPDA, TRX‐m4 + GSH, TRX‐f1/m4 + EtOH, t16: TRX‐h3 + OPDA, TRX‐h3 + GSH + OPDA, TRX‐h3/m4 + EtOH), *n* = 5 (all samples at t0, t1: TRX‐f1 + OPDA, TRX‐f1 + EtOH, t5: TRX‐m4 + OPDA, TRX‐h3/f1 + GSH, t16: TRX‐h3 + OPDA, TRX‐f1/m4 + GSH + OPDA, TRX‐h3/f1/m4 + GSH, TRX‐m4 + EtOH), or *n* = 6 (t1: TRX‐m4 + OPDA, TRX‐f1 + GSH, TRX‐h3 + EtOH, t5: TRX‐h3 + EtOH) independent replicates. OPDA and control samples were adopted from Fig. 5.

Additionally, insulin reduction activity of TRX‐h3, ‐f1 and ‐m4, pretreated with GSH, was analyzed after addition of OPDA (or 0.27% [v/v] EtOH as a control). Again, no effect of GSH on OPDAylation capacity could be observed (Fig. 11A–D). However, after prolonged incubation (16 h), reduction activity of TRX‐h3, ‐f1, and ‐m4 recovered to 89.7%, 93%, and 84.3%, respectively, indicating de‐OPDAylation.

**Fig. 12:**
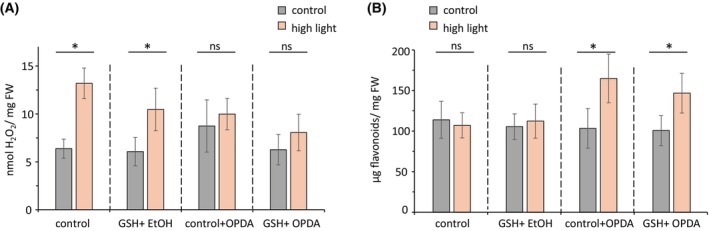
H_2_O_2_ and flavonoid contents of leaf discs exposed to excess excitation energy (EEE) stress for 6 h. Leaf discs were pretreated with 1 mm glutathione (GSH), or H_2_O as a control, for 16 h. Directly before stress treatment, 25 μm 12‐oxophytodienoic acid (OPDA) or 0.27% [v/v] EtOH was added to assess the effect of GSH pretreatment on OPDA signaling. A statistically significant increase (*P* < 0.05) in H_2_O_2_ or flavonoid levels after high light was calculated by t‐test and are denoted as *. Data are means ± SD of *n* = 5 (EtOH treatment at control conditions), *n* = 7 (GSH + OPDA at control conditions and EEE, GSH + EtOH at EEE) or *n* = 8 (all others) independent replicates. Mock‐treated samples correspond to those shown in Fig. 9 (panel C and D).

Lastly, the effect of GSH pretreatment was analyzed *in vivo* by applying 1 mm GSH to leaf discs and adding 25 μm OPDA after 16 h of incubation. After addition of OPDA, EEE was applied and contents of H_2_O_2_ and flavonoids were measured as described before (Fig. 9C,D). Neither OPDA‐dependent changes in H_2_O_2_ nor in flavonoid levels were disturbed by GSH pretreatment (Fig. 12A,B). This further supports the conclusions from the *in vitro* experiments that GSH does not scavenge OPDA before protein OPDA adducts can form.

### Retro‐Michael addition versus thiol exchange mechanism

The data shown so far mark OPDAylation as a reversible PTM, with possible release of bound OPDA moieties by excess low molecular weight thiols. However, the exact mechanism underlying de‐OPDAylation remains unclear. Hypothetically, adducts could either undergo *retro*‐Michael addition liberating OPDA, or OPDA could be transferred directly to another thiol‐containing compound by a thiol exchange mechanism that would then follow a nucleophilic substitution mechanism.

**Fig. 13:**
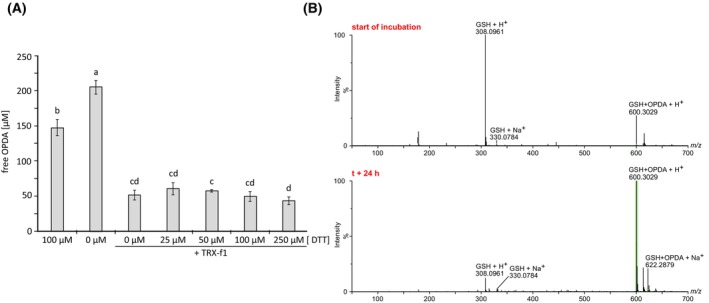
Quantification of 12‐oxophytodienoic acid (OPDA) after addition of dithiothreitol (DTT) to OPDAylated thioredoxin (TRX). 200 μm OPDA, pre‐incubated with buffer (left) or 50 μm TRX‐f1 (right) for 2 h, was treated with 0–250 μm DTT for 6 h prior to high pressure liquid chromatography (HPLC)‐based determination of free OPDA content (A). Data are means ± SD of *n* = 3 independent replicates; significant differences were assessed by one‐way ANOVA and Tukey test and are marked as a–d. Mass spectrometric analysis (B) corresponding to Fig. 3) was performed after addition of glutathione (GSH) to desalted TRX‐h3‐OPDA adducts over a time span of 24 h. Glutathione‐OPDA adduct (GS‐OPDA) was detectable from 4 h onward. After 24 h, free GSH + H^+^ and GS‐OPDA, but no OPDA could be detected.

To this end, levels of free OPDA were assessed after release of OPDA from TRX‐f1 (Fig. 13A) and TRX‐h3 (Fig. 13B). Neither the HPLC‐based quantification of OPDA nor the MS analysis showed a return of free OPDA. However, involvement of enzymes dedicated to de‐OPDAylation, for instance GSTLs or DHARs, as proposed before, might constitute an additional mechanism of OPDA release by recovering free OPDA.

## Discussion

OPDAylation has been proposed as a novel posttranslational modification regulating the activity of thiol‐containing proteins of the redox regulatory network [10]. Through this mechanism, OPDA influences redox homeostasis and subsequently gene expression *in planta* to promote stress defense. This study aimed at clarifying whether OPDAylation is a stable or reversible PTM by combining a variety of *in vitro* and *in vivo* studies.

### OPDAylation as a reversible process

The results of this study prove that OPDAylation of proteins is reverted in the presence of excess low molecular mass thiol compounds (GSH or DTT). The results show de‐OPDAylation for different proteins with established roles in the redox regulatory network, including the cytosolic TRX‐h3, plastid‐localized TRX‐f1 and ‐m4, and Cyp20‐3. De‐OPDAylation recovered the protein‐specific activity, namely the insulin reduction ability and electron donor capacity to GPXL8 as indicated by the H_2_O_2_ reduction (Figs. 5 and 6). In general, the extent of recovery increased in proportion to the protein's sensitivity to OPDAylation‐linked inhibition. For instance, PPIase activity of Cyp20‐3, which is highly sensitive towards OPDAylation, recovered by 154.1% after 4 h (Fig. 7C, at pH 7.2), while TRX‐m4 activity only increased by 12.6% (Fig. 5). Interestingly, like spontaneous GS‐OPDA formation, de‐OPDAylation was pH‐dependent (Fig. 7C). This might contribute to spatial regulation of de‐OPDAylation as adducts could be more stable in cell organelles with neutral pH (ER, cytosol, nucleus) compared to slightly alkaline compartments represented by the mitochondrial matrix, peroxisomes, and the chloroplast stroma under illumination [43].

**Scheme 1:**
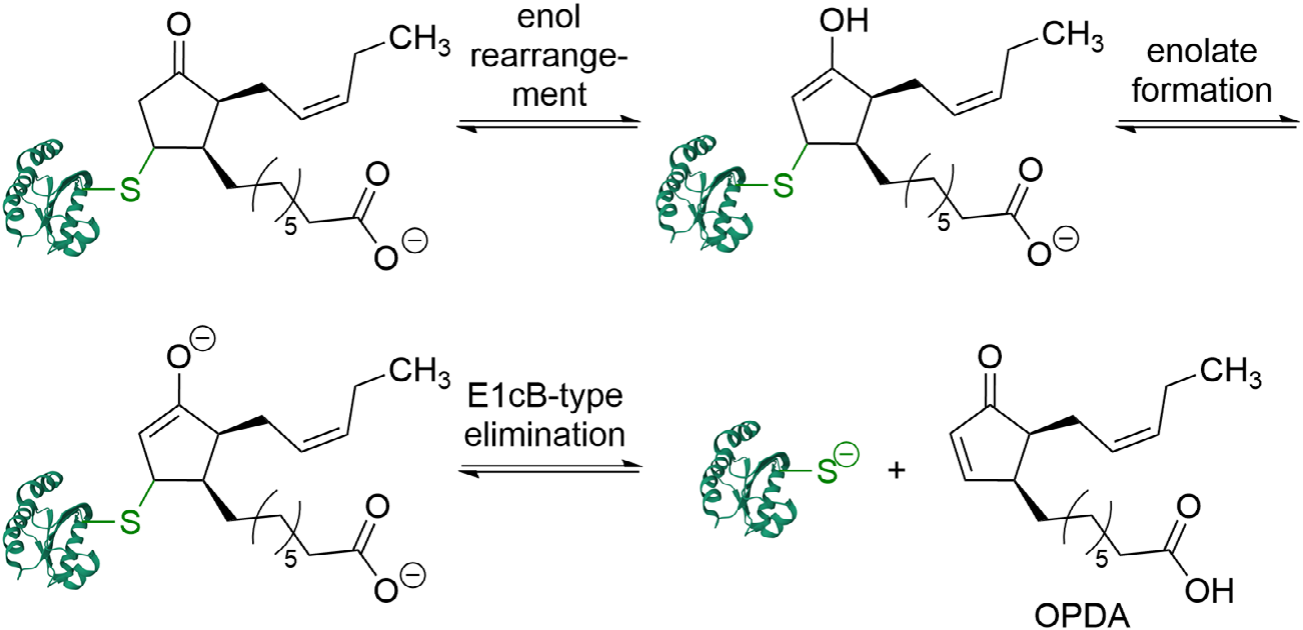
Proposed mechanism for the de‐OPDAylation of an 12‐oxophytodienoic acid (OPDA)‐adduct of thioredoxin (TRX). Cleavage of the OPDA adduct of thioredoxin proceeding through the steps of enol rearrangement, deprotonation, and subsequent first‐order elimination from conjugate base (E1cB)‐type elimination; protein data bank (PDB) reference of TRX‐h1 (from *Arabidopsis thaliana*; reduced form): 1XFL.

These findings are in accordance with a first‐order‐rate elimination from a conjugate base (E1cB mechanism), which has been reported for elimination processes of thioether products obtained from Michael addition reactions with thiols such as GSH and its replacement with methyl mercaptane [44]. The rate determining step in such an elimination is the formation of the enolate anion, and this step is favored under more basic conditions. Accordingly, a higher degree of de‐OPDAylation can be expected at an increased pH value, which is in agreement with the experimental observations described above. In analogy to the E1cB mechanism reported in literature for conversion of glutathione‐substituted Michael adducts to MeS‐substituted analogues [44], a proposed reaction mechanism is given in Scheme 1 for the de‐OPDAylation of the OPDA adduct with TRX and OPDA formation under release of the thiolate anion of TRX.

### Protein OPDAylation is favored over OPDA adduct formation with GSH

Under physiological conditions, the concentration of free thiols, for example, GSH, exceeds that of OPDA about 10^2^‐ to 10^3^‐fold. The fact that these thiols react with OPDA raises the question how basal levels of free OPDA are maintained for OPDA signaling as well as for processing to jasmonic acid. Remarkably, OPDAylation of TRX‐f1 and TRX‐m4 occurred *in vitro* independent on the presence of GSH, as indicated by OPDA‐dependent quenching of intrinsic Trp fluorescence and decreased insulin reduction activity (Tables 2 and 3, Fig. 11). *In vivo* studies on OPDA signaling during EEE further supported the occurrence of OPDAylation under physiological conditions (Fig. 12).

As depicted in Fig. 2, spontaneous GS‐OPDA formation is a slow process. For instance, after 4 h of incubation of 250 μm GSH and 500 μm OPDA at pH 7.2, ~ 50% of GSH was bound to OPDA. Further ^1^H NMR‐spectroscopic studies with an excess of GSH (ratio of GSH to OPDA of 5 : 1) support these findings of a relatively slow OPDAylation step with GSH as the thiol component as indicated by a conversion of 59% after 3 h when starting from a concentration of OPDA of 5 mm (Fig. 3). In contrast, protein OPDAylation proceeds rapidly, modifying the activity of target proteins distinctly already after 10 min [10, 12]. Different degrees of OPDA‐mediated inhibition of distinct proteins indicate specificity. Both factors appear to contribute to preferential binding of OPDA to protein thiols instead of GSH. However, various GSTs catalyze GS‐OPDA binding *in vivo* and will accelerate the reaction as discussed below [10, 11, 45], [34, 46].

**Scheme 2:**
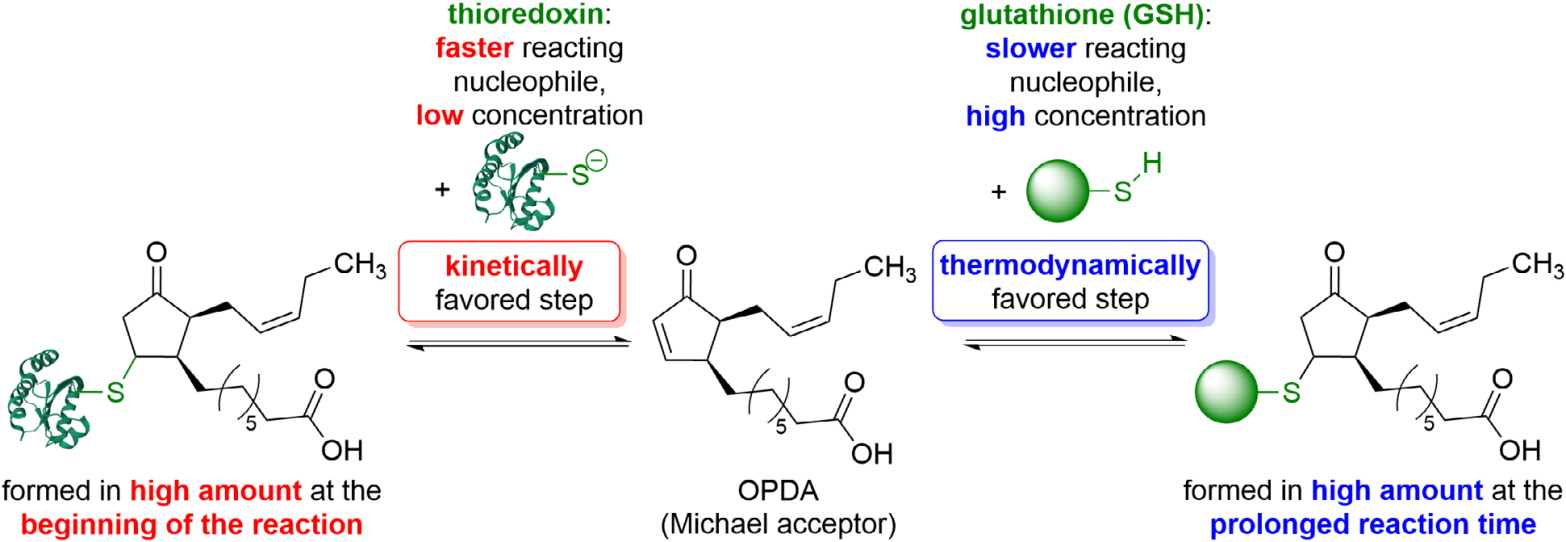
Kinetically versus thermodynamically controlled reaction for the formation of 12‐oxophytodienoic acid (OPDA)‐adducts of thioredoxin (TRX) and glutathione (GSH), respectively. Initially the preferred formation of the OPDA adduct of TRX prevails due to the fast, kinetically controlled addition of the thiolate in TRX and thermodynamically controlled formation of the OPDA adduct of GSH as the preferred adduct at prolonged reaction time due to the large excess of GSH; protein data bank (PDB) reference of the TRX (TRX‐h1 from *Arabidopsis thaliana*; reduced form): 1XFL.

These results can be rationalized by the hypothesis that initial TRX‐based OPDAylation is proceeding under kinetic control due to the higher reactivity of TRX compared to GSH. The higher nucleophilicity of proper TRX is due to the cysteine moiety C32 being present in TRX as a part of the active site of the reduced form of TRX, namely the Cys32‐Gly‐Pro‐Cys35 motif [47]. This molecular environment facilitates deprotonation of C32, which then reacts as a thiolate [47], thus leading to a much faster kinetically controlled Michael addition compared to the thiol moiety in GSH. However, due to the excess of GSH being present in the cell at much higher concentrations compared to TRX, at prolonged reaction time, the OPDAylation of GSH is observed as a result of a thermodynamically controlled process. The relationship of these two types of reversible OPDAylation reactions with the sulfur nucleophiles TRX and GSH, respectively, is visualized in Scheme 2.

### OPDA signaling under high light stress

The initial predominance of the OPDAylation process of certain proteins in the presence of GSH, followed by (slow) de‐OPDAylation, as observed *in vitro*, revealed novel dynamics of OPDA signaling. To scrutinize these findings *in vivo*, leaf discs pretreated with OPDA and/or GSH were subjected to EEE for 6 h followed by the determination of H_2_O_2_ and flavonoid content (Figs 9 and 11). OPDA positively regulates both the degree of ROS production and the cellular ROS detoxification capacity by stimulating the synthesis of antioxidants, chaperones, and photoprotective pigments in a Cyp20‐3‐dependent manner [11, 17, 21, 22, 23, 48].

**Fig. 14:**
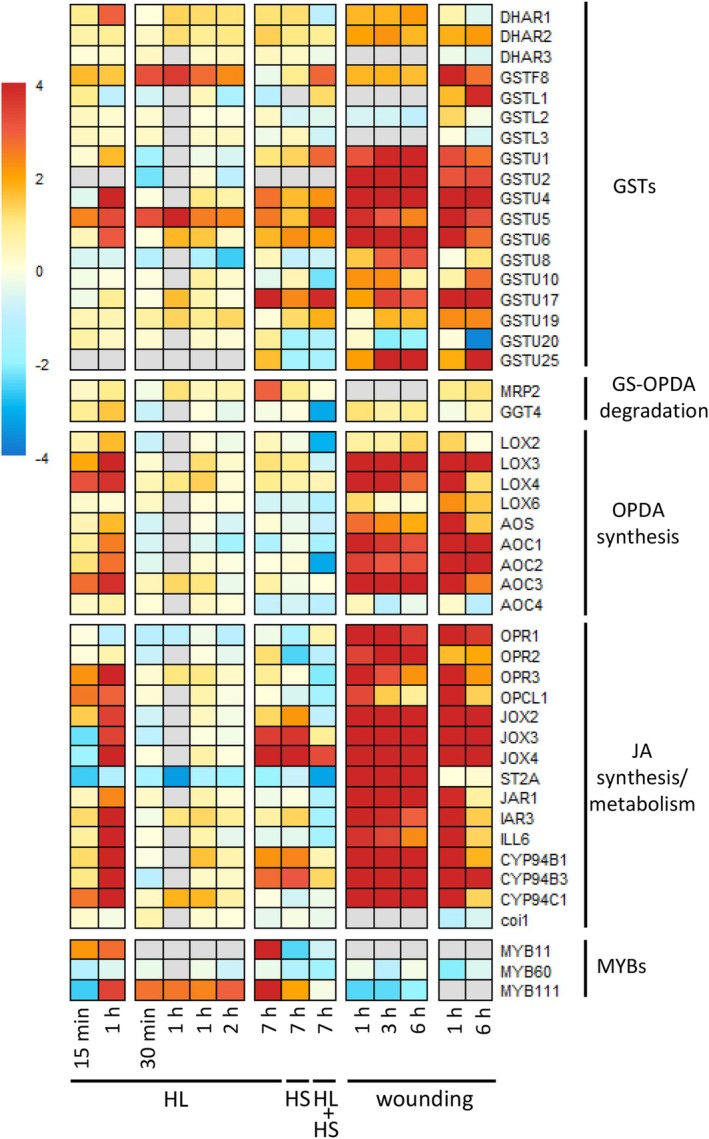
Expression profiles of selected genes after stress treatment. Values are presented as average LOG2FC at indicated time points (15 min–7 h). Data are based on publicly available transcriptome data from Kιlιç *et al*. (2025), Crisp *et al*. (2017), Balfagón *et al*., Xu *et al*. (2024), and Ikeuchi *et al*. (2017) (vertical sections from left to right). HL = high light, HS = heat stress, HL + HS = combined high light and heat stress.

Publicly available transcriptome data of *A. thaliana* subjected to EEE, heat stress or wounding (Fig. 14) reflects common upregulation of GSTs (and jasmonate synthesis) under nonoptimal growth conditions. However, affected GSTs seem to vary between stress type (and duration), creating specific GST signatures. For instance, GSTL1, GSTU2, and GSTU10 are more strongly upregulated after wounding, whereas DHAR3 and GSTU20 rather respond to high light stress together with MYB transcription factors MYB11 and MYB111. GSTU20, an identified target of OPDA signaling [45], as well as MYB11/MYB111 positively influence flavonoid synthesis and stability [49, 50, 51].

Leaf discs pretreated with 25 μm OPDA lacked the EEE‐induced increase in H_2_O_2_ observed in control samples. Instead, total flavonoid content was significantly enhanced, arguing for OPDA‐mediated acceleration of flavonoid biosynthesis (Fig. 12). Both readouts remained stable when leaf discs were pretreated first with GSH and secondly with OPDA. This result supports the preferential interaction of OPDA with protein thiols, specifically Cyp20‐3, previously observed *in vitro*.

However, de‐OPDAylation, as tested by addition of GSH after OPDA pretreatment, recovered only the EEE‐induced H_2_O_2_ accumulation, while flavonoid accumulation was not affected (Fig. 9). Although this appears contradictory at first, adjustment of flavonoid levels is much less dynamic compared to ROS levels, with first measurable increases after 12 –24 h or 10–60 min, respectively [19, 50, 52, 53]. Hence, modulation of H_2_O_2_ content can be regarded as an indicator of maintenance of OPDA signaling, while flavonoid levels display OPDA signaling in the early stage of EEE. Consequently, it might be assumed that excess external GSH did not impede initial OPDAylation of target proteins and resulting ORG expression, but the duration of OPDA signaling was shortened.

A possible reason might be a disturbance in the GS‐OPDA‐to‐OPDA ratio. GSTs accumulate already in early EEE stress [19]. Additionally, the stromal pH increases from pH 7 to more than 8 under illumination [54]. As both GSH binding and de‐OPDAylation showed a clear pH dependency, this might increase the GS‐OPDA‐to‐OPDA ratio in EEE further. Formation of GS‐OPDA (and detoxification of ROS) is GSH‐consuming, resulting in a decreased GSH‐to‐GSSG ratio. This decrease, and the simultaneous increase in GS‐OPDA, are measurable after 15 min of EEE stress [19]. As gene expression of MRP2 (EC:7.6.2.2) and GGT4 is not yet upregulated (Fig. 14), GS‐OPDA accumulation most likely serves as a means of OPDA transport and/or JA synthesis, and not degradation. Although JA content under EEE is not significantly enhanced in the long‐term [21], controlled conversion of OPDA to JA would allow for JA‐dependent stimulation of jasmonate synthesis. This would be in line with enhanced gene expression of OPR3 and OPCL1 (EC:6.2.1.‐) (Fig. 14) and minor increases in JA content, which are observable after 15–60 min [19]. The pool of reduced GSH available for GS‐OPDA formation might be a limiting factor at this point, preventing complete depletion of free OPDA. By supplementing GSH in the floating solution, however, this bottleneck might be avoided, resulting in increased turnover of OPDA and subsequent loss of OPDA signaling.

### Changes in cellular thiol composition modulate OPDA signaling

**Fig. 15:**
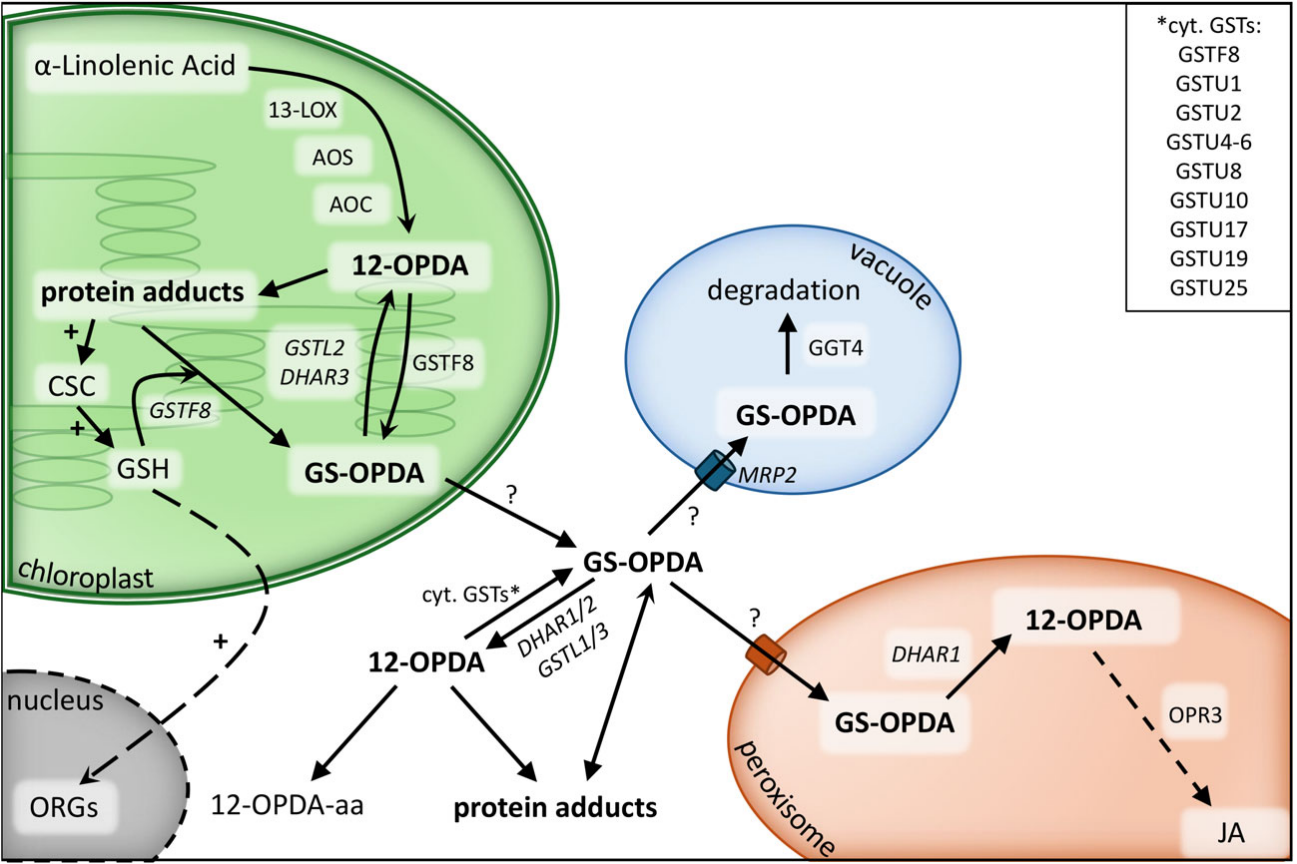
Availability of 12‐oxophytodienoic acid (OPDA) for signaling, degradation or conversion to jasmonic acid (JA) might be tightly linked to interaction with glutathione (GSH). OPDA generated in the chloroplast forms protein OPDA or GS‐OPDA adducts. While transfer of OPDA moieties between adducts is possible, free OPDA might be regenerated by dehydroascorbate reductases (DHARs) and glutathione‐S‐transferases of the lambda‐family (GSTLs). GS‐OPDA formation, transport, and regeneration of OPDA depend on expression pattern of GSTs. As interaction with cyclophilin 20–3 (Cyp20‐3) stimulates GSH synthesis and OPDA‐regulated genes (ORGs) expression, GST‐mediated OPDA modulation likely is a negative feedback system. Enzymes written in italics (GSTLs/DHARs) have not been analyzed for activity on GS‐OPDA so far.

Based on the interplay of protein OPDA adducts on the one hand and GS‐OPDA on the other hand, the following hypothetical scheme of OPDA signaling dependent on variations in cellular thiol composition might be deduced (Fig. 15).

Upon synthesis in the chloroplast, OPDA likely binds to proteins such as Cyp20‐3. The alkaline pH of the stroma established in the light may foster this reaction [55]. The adduct formation initiates OPDA signaling via activating the cysteine synthase complex and enhanced O‐acetylserine, Cys, and subsequently glutathione synthesis [56]. Considering the dynamic interplay between OPDAylation and de‐OPDAylation and the role of free thiols, the accumulation of GSH may be considered as feedback inhibitory circuitry ending or decreasing the OPDA‐dependent signaling in the chloroplast.

Free protein recovers by transfer of bound OPDA moieties to GSH, either spontaneously or, possibly, catalyzed by GSTs. While this transfer might occur in both directions, there is no regeneration of free OPDA in the spontaneous reaction (Fig. 13). However, unbound OPDA is crucial for JA synthesis, as GS‐OPDA cannot serve as substrate for OPR3 [57]. It may be hypothesized that GSTs of the DHAR and Lambda class, which can act as thioltransferases and show activity towards GS adducts [38, 58] catalyze this reaction. Like other GSTs, DHAR1 expression is regulated by OPDA and additionally by JA [17, 58].

Based on the subcellular localization of OPDA‐active GSTs in the cytosol and plastid stroma and GSTLs and DHARs in plastids, cytosol, peroxisome, and mitochondria, GST‐dependent modulation of the GSH‐OPDA interaction could serve as a means of OPDA transport. A similar mechanism of GSH‐mediated transport has already been described for animal prostaglandins [36, 59, 60]. Along this line, plastid OPDA could be bound to GSH by GSTF8 and transported into the cytosol (e.g., through ABC transporters) where it could target cytosolic proteins or be bound to amino acids after GSTL‐ or DHAR‐mediated adduct dissociation.

This hypothetical mechanism might control OPDA detoxification as a negative feedback system, as OPDA signaling causes upregulation of GST gene expression. With increasing GST levels, the GS‐OPDA‐to‐OPDA ratio likely increases as well. GS‐OPDA could then be transported either to the vacuole for degradation or to the peroxisome for OPDA release by DHAR1 and subsequent JA synthesis. Thereby, the dynamic interplay of OPDA with protein thiols and GSH might fine‐tune OPDA signaling, degradation, and metabolization.

## Conclusions

OPDAylation targets essential components of the plant redox regulatory network with severe functional implications. This observation motivated us to conduct this research with two main aims: First, OPDAylation was further scrutinized, and the results prove OPDAylation as a reversible PTM. De‐OPDAylation depends on the protein's sensitivity towards OPDA, the pH, and the concentration of excess thiol groups. This likely allows for precise modulation of OPDA amounts, as the rapid modification of protein activity enables subsequent induction of OPDA signaling, whereas de‐OPDAylation, triggered by increasing GSH, increasing cellular reduction potential (more negative redox potential), or enzymatically by GSTs, reduces oxidative damage which might be caused by pronounced alteration of the thiol switch proteins. It will be important in the future to explore the products of GS‐OPDA cleavage.

The involvement of GSH in protein recovery prompted the second focus of this work. Owed to its high abundance, GSH could act as an OPDA scavenger, modulating or preventing OPDA signaling and JA synthesis. However, OPDA signaling could be detected even in the presence of GSH. This interplay of OPDAylation and de‐OPDAylation might indicate a GSH‐dependent network regulating OPDA modification and signaling. GSH‐dependent modulation of OPDA signaling might contribute to the plant response to different stresses, especially those for which uncoupling of OPDA from JA signaling has been proposed [3, 19, 61, 62]. However, plant stress defense is highly complex and varies not only based on the type of stress, but also on other factors, in particular stress duration, stress intensity, developmental state of the plant, and on the combination of simultaneously occurring stresses [63]. Future studies should explore the involvement of DHARs and GSTLs in OPDAylation and de‐OPDAylation.

## Material and methods

### Expression and purification of recombinant proteins

Recombinant His‐tagged proteins were expressed in *E.coli* NiCo 21 (DE3) cells containing plasmids encoding for At3g62030 (Cyp20‐3), At1g63460 (GPXL8), At2g17420 (NTRA, EC:1.8.1.9), At5g42980 (TRX‐h3), At3g02730 (TRX‐f1), and At3g15360 (TRX‐m4), without transit peptides as described in [64], [10, 57], [12, 65]. Expression was induced by the addition of 400 μm isopropyl‐β‐D‐thiogalactopyranoside in the exponential phase and incubation at 37 °C for 4 h. Cells were harvested by centrifugation at 11 000 *x**g*** at 4 °C for 10 min, or, in the case of Cyp20‐3, at 4500 *x**g*** and 4 °C for 15 min.

After purification and dialysis, the purity of the proteins was assessed by Coomassie staining after separation in 12% (w/v) SDS/PAGE, and a Bradford assay (Roti®Quant, Roth, Karlsruhe, Germany) was conducted to determine the protein concentration.

### Synthesis of OPDA

12‐OPDA was provided and synthesized as described in previous literature. The spectroscopic data of the product were in accordance with the published data [66, 67].

### OPDAylation of thiol groups

Prior to OPDA treatment, 100 μm purified proteins were reduced by 10 mm DTT (RT, 1 h) and desalted using a PD10 column according to the manufacturer's instructions (gravity protocol, Merck, Darmstadt, Germany). 20 μm reduced protein was then incubated with indicated concentrations of OPDA or equal concentrations of EtOH as a control (RT, 16 h, if not specified otherwise).

### Mass spectrometric analysis of OPDAylated thiols

#### MS analysis of protein thiols

MS analysis was performed by applying 10 μm protein (in 50% ACN+ FA) to a Q‐IMS‐TOF mass spectrometer Synapt G2Si (Waters GmbH, Manchester, UK) operated in resolution mode and interfaced to a nano‐ESI ion source. N_2_ generated with the nitrogen generator NGM 11 served as nebulizer and dry gas. Argon was used as collision gas for MS2 experiments using collision‐induced dissociation (CID). Samples were introduced by static nano‐ESI using in‐house pulled glass emitters. External calibration of the mass axis used ESI‐L Tuning Mix (Agilent Technologies, Santa Clara, CA, USA) as standard. Scan accumulation and data processing were performed with MassLynx 4.1 (Waters GmbH, Manchester, UK) on a PC workstation. The final spectra were generated by accumulating and averaging 50 single spectra. Determination of exact masses of OPDA was performed based on centroided data from the protonated signal of leucine‐enkephalin as an internal mass standard. Determination of protein masses was performed with the tool of Winkler [22]. Prior to protein mass determination, MS data were baseline subtracted, smoothed, and centroided.

To test different OPDA:TRX ratios, TRX‐h3 was prepared as specified above and eluted in 50 mM ammonium acetate. 20 μm reduced TRX‐h3 was treated with 20–80 μm OPDA (or 0.07–0.27% [v/v]) EtOH overnight and then subjected to MS analysis. For stability analysis of OPDAylated TRX‐h3, 20 μm TRX‐h3 was incubated with 80 μm OPDA overnight, then desalted (Zeba Spin Desalting Columns 7 K MWCO, Thermo Scientific, Darmstadt, Germany) to remove excess OPDA and applied to MS after 0, 3, 6, or 24 h. De‐OPDAylation assays of TRX‐h3 were performed in the same manner; however, after desalting, 1 mm GSH was added to the sample.

#### MS analysis of nonprotein thiols

Mass spectrometry was performed analogously to 2.4.1, with minor adjustments: 150 μm OPDA were incubated with 1 mm GSH or DTT (in 50 mm ammonium acetate) overnight and either analyzed directly or, for competition assays, 1 mm thiol compounds as indicated was added, and the presence of the OPDA adducts was analyzed before and directly after addition, as well as after 1, 2, 4, or 24 h.

### H‐NMR‐spectroscopic monitoring of GS‐OPDA‐formation

^1^H‐NMR‐spectroscopic measurements were performed on a Bruker Avance III 500 and a Bruker Avance III 500HD. The solvent signal used for the internal calibration of the NMR spectra was D_2_O (δ = 4.79) [68]. For the NMR experiment, OPDA (1.2 mg, 0.004 mmol, 1 eq.) and GSH (6.3 mg, 0.020 mmol, 5 eq.) were dissolved in D_2_O (800 μL). To increase the solubility of OPDA, the pH of the solvent was adjusted to pH 8 using NaOH. The sample was incubated at RT and measured after 10, 140, and 180 min.

### Quantification of free thiol groups

250 μM of DTT or GSH was incubated with 500 μm OPDA (or 0.56% [v/v] EtOH as control) in 40 mm KPi buffer of different pH values (pH 5.7–8.7) for 30 min at 750 rpm shaking speed and 25 °C. Residual free DTT or GSH was determined by 5,5′‐dithiobis‐2‐nitrobenzoic acid (DTNB) assay using a standard curve of 0–500 μm GSH [12]. Samples were measured at 412 nm with a plate reader (KC4, BIOTEK Instruments).

### Insulin reduction assay

TRX‐mediated insulin reduction was performed as described in [10]. Briefly, 20 μm TRX‐h3, TRX‐f1, or TRX‐m4 were incubated with 80 μm OPDA or EtOH (0.27% [v/v] as control) at RT for 1 h with subsequent addition of 1 mm GSH or equal volume of water as a control. To study the effect of GSH pretreatment, protein was first incubated with GSH or water followed by addition of OPDA or equal EtOH concentrations as control. To measure insulin precipitation (in a total volume of 200 μL), 5 μm of treated protein was added to 100 mm KPi, pH 7.2, 1 mm EDTA, and 160 μm insulin in a 96‐well plate. After measuring baseline absorbance, DTT (final concentration of 1 mm) was added, and absorbance was recorded at 650 nm for 60 min.

### TRX‐dependent peroxidase activity

TRX activity was measured as electron donor to glutathione peroxidase‐like 8 (GPXL8) after addition of H_2_O_2_ according to [10]. Briefly, 20 μm of reduced TRX‐h3 was incubated with 150 μm OPDA (or 0.5% [v/v] EtOH) for 1 h at RT. Next, 0.25–5 mm GSH and 0.25 μm TRX were added to a reaction mix containing 200 μm NADPH, 1 μm NADPH‐dependent thioredoxin reductase A (NTRA), and 3 μm GPXL8 (in 0.1 m Tris/HCl, pH 7.5, 5 mm EDTA). After recording the baseline for 3 min, addition of 300 μm H_2_O_2_ started the enzyme assay. The consumption of NADPH+H^+^ by NTRA was monitored at 340 nm (Cary 3500 UV–Vis, Agilent, Santa Clara, CA, USA).

### Cyp20‐3 activity

PPIase activity of Cyp20‐3 was measured spectrophotometrically as described in [12]. Reduced Cyp20‐3 (5, 10, 25, 50, 75, 100 nm) was mixed with 100 μm N‐succinyl‐Ala‐Ala‐Pro‐Phe‐p‐nitroanilide and either 25 μm OPDA, 0.083% [v/v] EtOH, 1 mm DTT or 1 mm H_2_O_2_ in 35 mm HEPES, pH 8.0 in polystyrene cuvettes (total volume of 2 mL). The sample mix was incubated at 8 °C and 800 rpm for 10 min. After recording the baseline at 390 nm for 3 min (Cary 3500 UV–Vis, Agilent, Santa Clara, CA, USA), the reaction was started by adding 0.4 mg·m^−1^ α‐chymotrypsin.

De‐OPDAylation of Cyp20‐3 by GSH was analyzed by first incubating 20 μm of reduced Cyp20‐3 with 150 μm OPDA (50 mm HEPES, pH 8, RT, 2 h), followed by addition of 1 mm of GSH and further incubation (48 h at RT). For pH dependency, incubation conditions were modified to 40 mm KPi adjusted to pH 6.2, 6.7, 7.2, 7.7 or 8.2 and 4 h of incubation at RT.

### Tryptophan fluorescence

15 μm of reduced TRX‐f1 or TRX‐m4 (dissolved in 50 mm KPi, pH 7.2) was incubated with 150 μm OPDA, 0.5% [v/v] EtOH, 1 mm H_2_O_2_ or 1 mm DTT for 3 h. For de‐OPDAylation, samples were further incubated for 16 h after addition of 1 mm GSH or DTT. To assess the influence of GSH incubation prior to OPDAylation, 15 μm reduced protein was first incubated with 1 mm GSH for 2 h, followed by addition of 150 μm OPDA (or 0.5% [v/v] EtOH as a control) and 3 h of incubation. All steps were performed at RT.

Relative tryptophan fluorescence was determined using a spectrofluorometer (SFM 25, Kontron Instruments), set to an excitation wavelength of 280 nm, a scan rate of 1 nm·sec^−1^, high voltage 450 V and an emission wavelength range of 300–400 nm.

### HPLC‐based quantification of OPDA

Quantification of OPDA was performed as described in [10]. 200 μm OPDA was incubated with 50 μm TRX‐f1 in 40 mm KPi pH 7.2 at RT for 2 h. 25–250 μm DTT was added and the residual amount of free OPDA was measured after 4 h by applying 25 μL of sample to a Dionex HPLC system equipped with a LiChrospherR 100 RP‐18 column (LiChrom‐CARTR 250–4, Merck, Darmstadt, Germany) set to 30 °C and 1.75 mL min^−1^. After 9 min of equilibration with solution A (80% [v/v] MeOH, 0.1% [v/v] acetic acid), solution B (99.9% [v/v] MeOH, 0.1% [v/v] acetic acid) was gradually added (9–14 min 0–25% solution B, 14–16 min 25–100% solution B). Each run was recorded at 224 nm (Chromeleon Version 6.6, Dionex) for 18 min. A standard curve of 0–250 μm OPDA was used to calculate the concentration of OPDA in the samples.

### Plant growth and preparation of leaf discs

#### Initial setup

*A. thaliana* Col‐0 and *coi1* (kindly provided by Dr. Roberto Solano) plants were grown in soil in a climate chamber with a day/night cycle of 10h/ 14h with 80 μmol photons m^−2^·s^−1^, 21 °C/14 °C and 50% relative humidity. Prior to that, seeds were incubated at 4 °C for 3 days. After 6–7 weeks of growth, leaf discs of 5 mm diameter were cut out using a cork borer and immediately placed upside down in a 96‐well plate filled with either 25 μm oxylipin solution (containing 0.1 mm CaCl₂) or, as a mock control, 0.083% [v/v] EtOH solution (also containing 0.1 mm CaCl₂). After 16 h of incubation, leaf discs were subjected to high light stress (800 μmol photons m^−2^·s^−1^) for 6 h and frozen in liquid nitrogen for determination of H_2_O_2_ and (dehydro‐) ascorbate content.

#### Final setup

For the final test system, growth and treatment conditions were slightly modified. Leaf discs were prepared from 35 to 40 days old *A. thaliana* Col‐0 plants grown in soil in a greenhouse (day/night cycle of 8 h/16 h, 100 μmol photons m^−2^·s^−1^, 22 °C/20 °C, 45–55% relative humidity). Floating solutions were prepared without CaCl_2_ and prior to EEE treatment, 1 mm GSH (or H_2_O as a control) was added. To analyze OPDAylation effects after GSH pretreatment, leaf discs were first incubated with 1 mm GSH for 16 h and 25 μm OPDA (or 0.083% [v/v] EtOH) was added directly before EEE treatment. H_2_O_2_ and flavonoid levels were chosen as final readouts of OPDA signaling.

### Measurement of H_2_O_2_ content

H_2_O_2_ content of *A. thaliana* leaf discs was determined according to [69] with minor modifications. About 35–45 mg finely ground plant material (8 leaf discs shock‐frozen in N_2 liquid_) was homogenized in 0.5 mL of 5% (v/v) trichloroacetic acid at 4 °C and then incubated in the dark at 4 °C for 40 min. To remove proteins, samples were centrifuged (10 000 *x**g***, 15 min, 4 °C) and 50 μL of the supernatant was diluted 250‐fold in 0.1 m sodium carbonate buffer, pH 10.2. Then, 20 μL of dilution was mixed with either 5 μL 50 U catalase (EC:1.11.1.6) or 5 μL H_2_O and incubated in the dark at 30 °C for 15 min. Chemiluminescence was measured at 425 nm for 5–8 s after adding 2 μL of sample (catalase‐treated or untreated) to 998 μL working solution (65 μm luminol, 30 μm CoCl_2_ in 0.1 m sodium carbonate buffer, pH 10.2). A standard curve was generated with H_2_O_2_ (0–15 μm).

### Measurement of flavonoid content

Flavonoid content of leaf discs was determined based on [70] with several adjustments. 100 μL of plant extract (prepared as described in 2.12) was mixed with 400 μL of H_2_O and 30 μL of 5% (w/v) NaNO_2_ and incubated at RT for 6 min. 30 μL of 10% [w/v] AlCl_3_ was added and the reaction was stopped after 6 min by adding 400 μL 1 m NaOH. The volume was adjusted to 1 mL with H_2_O. After 15 min, 150 μL of sample was transferred to a microtiter plate and OD_405 nm_ was measured using a plate reader. Flavonoid content was calculated based on a standard curve of 0.5–10 μg quercetin.

### Measurement of ascorbate and dehydroascorbate

The ascorbate content of leaf discs was determined using a plate reader according to method used in [71] with minor modifications. About 10 mg of leaf discs were extracted in 100 μL of HCl (0.2 N), and the extract was neutralized with 0.2 m NaOH. The sample mixture composed of 45 μL of the neutralized extract, 57.9 μL of 0.12 m sodium phosphate buffer (pH 7.5), and 4.3 μL of 25 mm DTT (for total ascorbate) or H₂O (for reduced ascorbate). Total and reduced ascorbate contents were determined based on the decrease in absorbance at 265 nm using 5 μL of ascorbate oxidase (0.05 U·μl^−1^). The amount of dehydroascorbate was found by taking the difference between total ascorbate and reduced ascorbate. The calculations were performed using a corrected extinction coefficient of 7000 M^−1^·cm^−1^.

### Transcriptome analysis

To analyze differential expression of selected genes under high light, heat or wounding stress, RNAseq data were obtained from the NCBI GEO repository and iDEP.96 was used for identification of differential gene expression [72]. Data sets used were GSE277977 [19], GSE101422 [73], and GSE134391 [21]. Additionally, transcriptome data generated by Crisp *et al*. (2017) and Xu *et al*. (2024) were included [74, 75].

### Statistical analysis

To determine significant differences, either one‐way ANOVA in combination with *post hoc* Tukey, Dunnett, or *t*‐test with Bonferroni correction were carried out using RStudio (2025.05.0 + 496) [76].

## Author contributions

Conceptualization, AV, HG, MK, and K‐JD; methodology and investigation, AK, JS, LV, MB, MK, and RS; resources, HG and M.B; writing—original draft preparation, MK; review and editing, all authors; supervision, K‐JD; funding acquisition, HG and K‐JD. All authors have read and agreed to the published version of this manuscript.

## Data Availability

All original data supporting the findings of this study are available within this paper.
